# Avian Incubation Patterns Reflect Temporal Changes in Developing Clutches

**DOI:** 10.1371/journal.pone.0065521

**Published:** 2013-06-19

**Authors:** Caren B. Cooper, Margaret A. Voss

**Affiliations:** 1 Bird Population Studies, Cornell Lab of Ornithology, Ithaca, New York, United States of America; 2 School of Science, The Pennsylvania State University at Erie, Erie, Pennsylvania, United States of America; CNRS, Université de Bourgogne, France

## Abstract

Incubation conditions for eggs influence offspring quality and reproductive success. One way in which parents regulate brooding conditions is by balancing the thermal requirements of embryos with time spent away from the nest for self-maintenance. Age related changes in embryo thermal tolerance would thus be expected to shape parental incubation behavior. We use data from unmanipulated Black-capped Chickadee (*Poecile atricapillus*) nests to examine the temporal dynamics of incubation, testing the prediction that increased heat flux from eggs as embryos age influences female incubation behavior and/or physiology to minimize temperature fluctuations. We found that the rate of heat loss from eggs increased with embryo age. Females responded to increased egg cooling rates by altering incubation rhythms (more frequent, shorter on- and off- bouts), but not brood patch temperature. Consequently, as embryos aged, females were able to increase mean egg temperature and decrease variation in temperature. Our findings highlight the need to view full incubation as more than a static rhythm; rather, it is a temporally dynamic and finely adjustable parental behavior. Furthermore, from a methodological perspective, intra- and inter-specific comparisons of incubation rhythms and average egg temperatures should control for the stage of incubation.

## Introduction

The quality of incubation influences reproductive success in species that care for their eggs. Brooding conditions affect avian embryo growth, survival, hatchling weight, and subsequent offspring behavior and fecundity [1] [2] [3] [4]. The type of parental care of eggs, and the subsequent degree of parental influence on pre-hatch conditions, varies greatly among species [5]. Female-only incubation has been documented in 62% of passerines species [6]. Female-only incubation is generally assumed to begin with partial incubation [7] with an increase to fully developed, although intermittent, incubation at some point near the end of the egg-laying period. As such, most passerine eggs undergo periodic cooling, which can reduce embryo growth efficiency [8] and increase parental energy expenditure during incubation [9] [10].

Prior to clutch completion and during the transition from partial to full intermittent incubation, behavioral patterns vary widely [7]. Full incubation is thought to occur when nest attentiveness reaches a threshold [11], after which females settle into a pattern of relatively high attentiveness until hatch [12] [13]. In small passerines, this pattern is likely to reflect a balance between the time and energy allocations required to support both parental self-maintenance activities and incubation [14] [15] [16] [17].

The generalization of a fairly static intermittent attentiveness pattern, however, does not account for the possibility that embryos of different ages vary in thermal tolerance, or that parental time and energy investments may change over time [18]. Incubation directly increases the energy expenditure of incubating birds [19] [9] [20]. This increase in energy expenditure must be balanced with other demands and the finite time available for parents to acquire the food needed for energy production. The adjustments incubating adults make to balance their needs and their embryo's needs are expressed through their incubation rhythms – the durations of time spent continuously on their nests (on-bouts) and the frequencies and durations of periods spent away from their nests (off-bouts). While there are many important studies concerning the intermittent incubation behavior of wild birds (e.g., [15] [21] [22] [23] [13]), most of our knowledge about the pace and mechanics of avian embryo development comes from studies of domesticated fowl (*Gallus gallus*) [18] [24] [25]. It is unclear that studies of galliform species, with precocial offspring and nest attendance of more the 95% [6], are relevant to understanding the relationship between altricial embryo development and the relatively low nest attendance of intermittent incubators. The data presented in this paper represent a step in that direction.

We used detailed nest temperature measurements of an intermittent incubator, the Black-capped Chickadee (*Poecile atricapilla*), to examine the dynamic relationship between embryo development and parental incubation. We focused on two properties of embryos that we hypothesized are age-dependent: cooling rate (circulation) and thermal tolerance. Specifically, we hypothesized that, as embryos age, increased egg-cooling rate results in changes in female incubation (behavior and/or physiology) which, in turn, adjusts eggs temperatures to meet decreasing thermal requirements. Our approach was to determine whether patterns in the wild were consistent with our hypotheses.

First, following reductionist biophysical models of heat flow [26] [27], we predicted that the rate of heat loss from contact-incubated eggs will increase as embryos develop. Early in incubation, heat flows through eggs only by conduction, which results in low heat loss. Later in incubation, there is axial transport of heat due to embryo circulation, which increases the loss of heat [26] [27]. For example the thermal conductance of chicken eggs increased 20% as a function of increased blood flow [28]. Also increased heat conductance should increase the overall energy costs of incubation [26] [27]. Embryonic circulation for an egg of domesticated fowl (*G. gallus*) approximately doubled the parents' energy cost of maintaining egg temperature [29]. Second, we predicted that the thermal tolerance of embryos decreases as they develop. Birds progress along a trajectory from ectothermy to endothermy as they develop in the egg and nest [30]; as a consequence, they have higher and narrower thermal requirements as they grow [18].

If there are increases in cooling rates and thermal requirements as embryos develop, then we predicted temporal dynamics in incubation, specifically that females will respond to minimize fluctuations in egg temperatures: physiologically with vasodilation to increase brood patch temperature and/or behaviorally with adjustments in the duration and frequency of off-bouts. Indeed, there is evidence that parental incubation behavior can be influenced by signals (e.g., vocalizations or movement) from late stage embryos in response to changes in thermal conditions [31]. Furthermore, it is clear that embryo heart rate is substantially altered by changes in ambient temperature, even late in embryo development [32].

Thus, with an observational, field approach, we addressed the following questions: (a) does daily average egg-cooling rates increase over the course of incubation?; (b) do average daily cooling rates covary with daily incubation behaviors and/or physiology?;, and (c) What can we infer about thermal tolerance over the course of incubation?

## Methods

### Study species

Our study was carried out near Erie, Pennsylvania (42°07′N, 80°05′W) in eastern deciduous forest containing wooden nest boxes occupied by nesting black-capped chickadees. Black-capped chickadees were useful for this study because they are small (9–14 g) intermittent incubators that tolerate nest disturbances such as the placement of fake eggs to measure temperatures. Black-capped chickadees produce a range of clutch sizes (4–9 eggs) [33] with a reported mean of “just under 7 eggs” [34]; the 21 clutches we observed were consistent with these values (6.3±1.46 eggs [SD], n = 21 clutches). We grouped clutches into three size classes: small (≤4 eggs, n = 3), intermediate (6 eggs, n = 11), and large (8 eggs, n = 7). We did not artificially manipulate clutch size. Naturally occurring small clutches are exceedingly rare, thus we have a small sample size for that category.

### Field methods

We recorded egg temperatures and estimated female incubation rhythms throughout the incubation period in 21 black-capped chickadee nests with first egg dates that ranged from 29 April to 30 June each year (2003–2006). For each nest, we used a miniature battery powered dual-channel ambient temperature and remote thermocouple data logger (OM-CP-TC4000, Omega Engineering Inc.) placed under nests to record ambient box temperature. To measure egg temperatures, we attached to the remote port of each data logger a factory welded 36-gauge constantan-chromega thermocouple (Omega Engineering Inc, Stamford Connecticut) implanted in silicon gel (Ideal Industries, Sycamore, Illinois) filled plastic craft eggs (1.5 cm×1.2 cm) that resembled black-capped chickadee eggs in both size and coloration. The thermocouple wire was woven through the nest material so that each egg was anchored in the center of the nest cup; in all cases, the real eggs circled the anchored thermocouple egg. Similar methods have previously been used to investigate egg cooling rates in other bird species [2] [35]. However, we used thermocouples instead of thermistor thermometers because thermistors release heat while in use and therefore bias the temperature recordings of small eggs. For each nest, both box temperatures and egg temperatures were recorded simultaneously every 15 s during the incubation period.

Measuring the temperature in a silicon-filled egg in the center of the nest cup is a highly conservative way to estimate clutch-cooling rates because the thermal properties of the silicon gel do not change over the course of incubation. Consequently, any systematic differences in measured egg temperatures were driven by the cooling properties of the surrounding eggs in the clutch. According to Turner's model, early in incubation each egg loses heat from the surface where the heat was applied in contact incubation. Later in incubation the embryo in each egg transports heat to all egg surfaces so that heat is lost in areas where the egg is in contact with the thermocouple egg, as well as to the air or brood patch.

All observations and measurements were made under an approved Institutional Animal Care and Use Committee protocol administered by The Pennsylvania State University (IACUC # 16175) and bird banding permit # 23280 issued to M. Voss from the United States Geological Survey's Bird Banding Laboratory.

### Data reduction

We used temperatures of silicon-filled eggs throughout each 24 hrs to estimate daily mean egg temperatures, and the minimum and maximum egg temperatures associated with each off-bout, and to infer female incubation behaviors. We used the software combination of Rhythm and Raven [36] to graphically display temperature fluctuations over time and measure the duration and frequency of all on-bouts and off-bouts, inferred by the temperature fluctuations. We used criteria of a minimum decrease in egg temperature of 3.0 C, a minimum initial cooling slope of 0.3 C/min and minimum off-bout duration of 3 minutes in Rhythm to delineate off-bouts from the nest. The Rhythm output for each temperature record was visually inspected in Raven to verify both incubation off-bouts and on-bouts.

We back calculated embryo age from the day of hatch and report values in days prior to hatch. We obtained sufficient samples to analyze data from day of hatch to 14 days prior to hatch. We measured nest attentiveness during daylight hours and report percent time off the nest as Σ (off-bout durations/day)*100. Mean daily T_egg_ was the mean egg temperature during the active day, including both off-bout and on-bout periods, where active day is equal to the duration between the start of the first off-bout of a day and the end of the last off-bout.

In order to broaden our inference regarding the mechanism by which females influence egg temperature, we sub-sampled when ambient temperatures were <25°C and prior to 1200 hr for computing egg-cooling rates. We examined female incubation off-bout durations in relation to corresponding cooling rates during that off-bout and minimum egg temperature during that off-bout.

Also from the sub-sample of cooler morning hours, we estimated brood-patch temperature as the asymptote of the log-normalized proportional increase in temperature during each incubation on-bout [37] using the regression method [38]; this technique was developed to determine body temperatures of ectotherms at equilibrium with their environment. Eggs are functionally ectothermic and reach a thermal equilibrium during incubation, thus the asymptote of the heating function is brood patch temperature. It was necessary to identify egg heating rate constants (min^−1^) for the calculation of brood patch temperature.

### Statistical analyses

In all analyses using general linear mixed models (GLMM), we used day of incubation nested in nest identity as a repeated factor. In all GLMM analyses, clutch size and embryo age were categorical variables, any interactions that were not significant were removed, and we used the Satterthwaite method to estimate denominator degrees of freedom. We fit all GLMM with restricted maximum likelihood, and the structure of the repeated effect variance was modeled as a variance components structure (GLMM; SAS PROC MIXED) [39].

### Does daily average egg-cooling rate increase over the course of incubation?

We used GLMM to model egg-cooling rate as a function of embryo age, clutch size, and the interaction (all categorical variables).

### Do average daily cooling rates account for variation in daily incubation behaviors and/or physiology?

We used GLMM model the behavioral components of nest attendance as averages per day (e.g., mean number of off-bouts, mean duration of off-bouts, and mean duration of on-bouts) as functions of egg-cooling rate, embryo age, clutch size, and the interactions of clutch size with each of the other variables. We used GLMM to describe the relationship between female nest attendance as a function of its behavioral components: number of off-bouts per day, mean duration of off-bouts, and mean duration of on-bouts. We arc-sine-square-root transformed the percent time off the nest. We log-transformed the mean duration of daily off-bouts. We back-transformed estimates of these variables when reporting results.

We also used GLMM to model estimated brood-patch temperature as a function of egg-cooling rate, with covariates of mean daily off-bout duration and mean daily on-bout duration.

### What can we infer about embryo thermal tolerance over the course of incubation?

We used simple linear regressions to model the coefficient of variation in egg temperature, daily and nightly, as a function of embryo age. We used GLMM to model daily egg temperatures (the mean, maximum, minimum) as functions of embryo age, clutch size, and their interaction.

## Results

The mean incubation period for the 18 nests that hatched was 12.89±0.8SD. Three nests were lost to predators after 11 days of incubation.

### Do daily average egg-cooling rates increase over the course of incubation?

**Figure 1:**
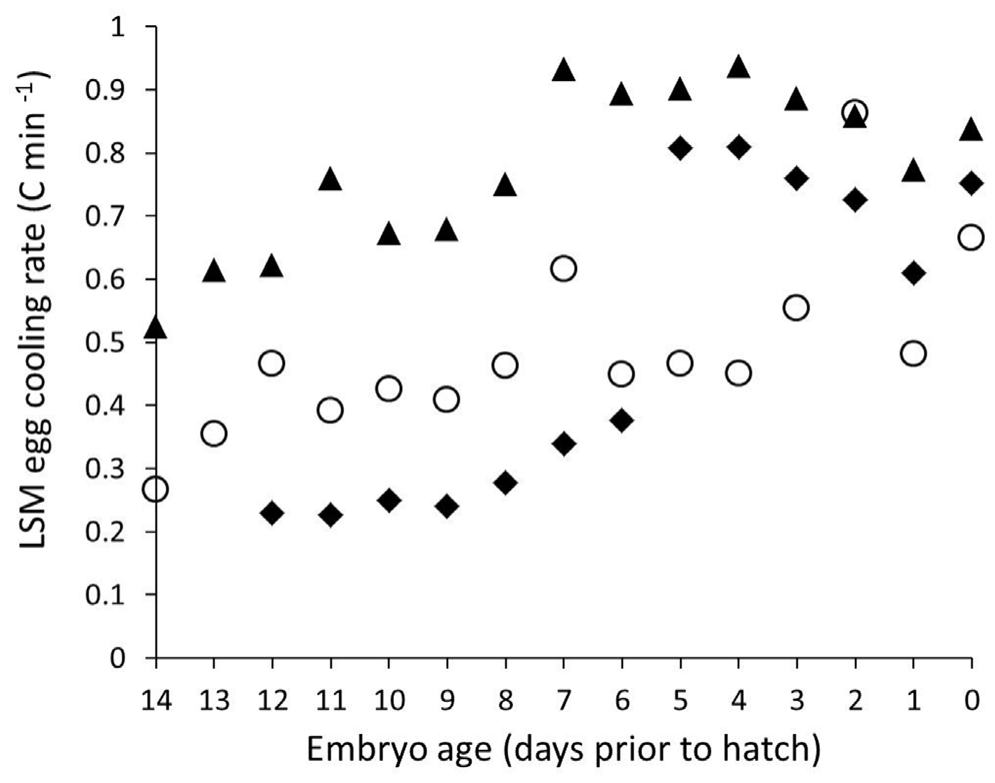
Egg-cooling rates during incubation off-bouts. Least squares mean estimates (± SE) of egg-cooling rates (°C min^−1^) increased as embryos aged for naturally incubated eggs in 21 black-capped chickadee nests for clutches of size 3 (diamonds), 6 (triangles), and 8 (circles).

The daily mean rate of egg cooling increased as embryos approached hatch, and the relationship varied with clutch size such that eggs in small (4-egg) clutches had higher cooling rates than eggs in larger (6- or 8-egg clutches) (GLMM *F* = 2.14, df = 1752, *P*<0.0007, Fig. 1)

### Do average daily cooling rates account for variation in daily incubation behaviors and/or physiology?

**Figure 2:**
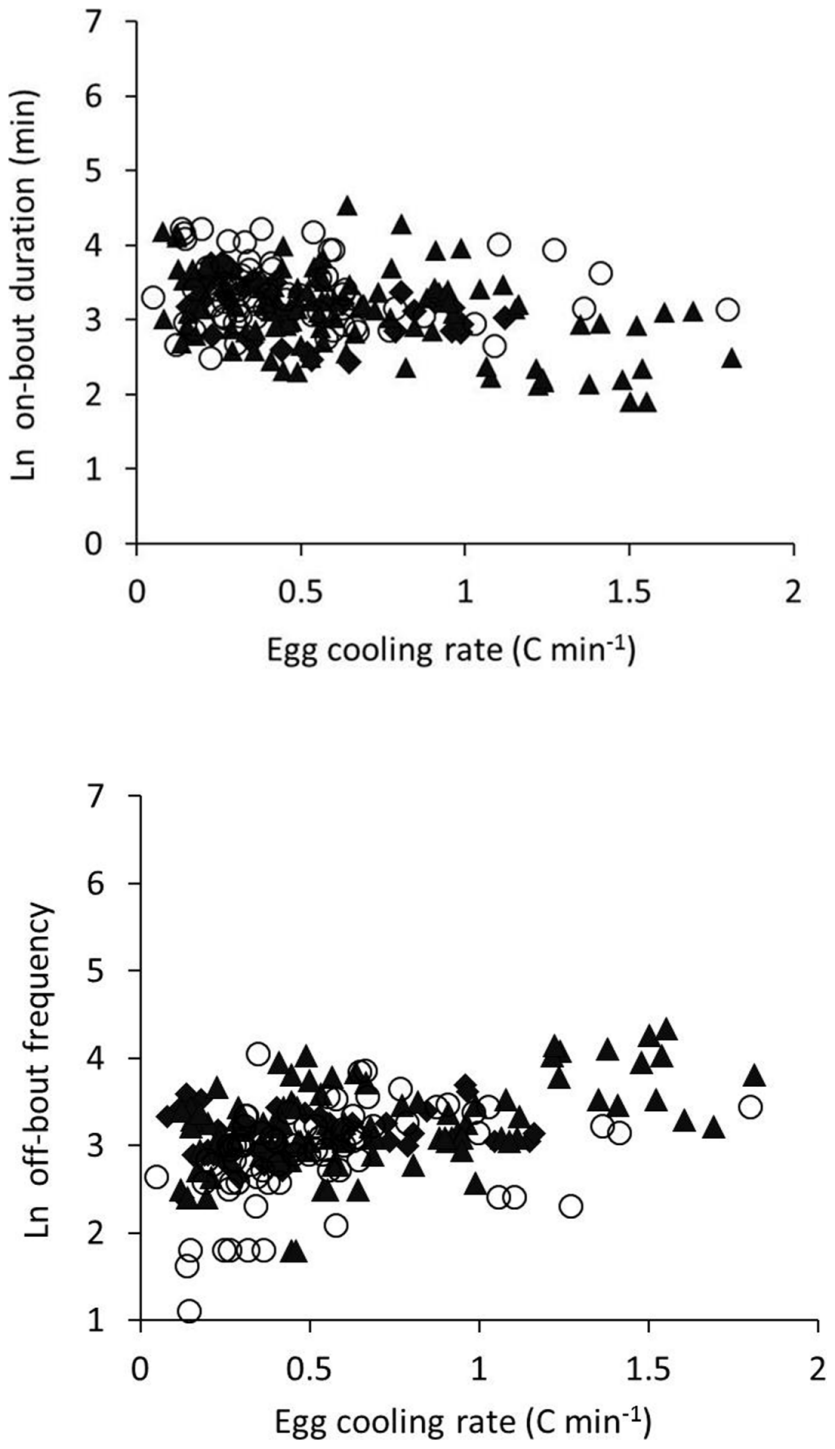
Off-bout frequency and on-bout duration as functions of egg-cooling rate. Log-normalized number of daily off-bout duration (bottom) and mean daily on-bout duration (top) for 21 clutches of black-capped chickadees as functions of mean daily egg-cooling rate (°C min^−1^). The number of daily off-bouts increased and on-bout duration decreased as clutch cooling rates increased.

The frequency of off-bouts increased as egg-cooling rate increased, particularly for small clutches (GLMM: *F = 4.13*, df = 215, *P* = 0.004; Fig. 2). Off-bout duration decreased as embryos aged (F = 31.69, df = 216, P<0001), and did not vary with egg-cooling rate (F = 0.54, P = 0.5) or clutch size (F = 2.21, P = 0.1). On-bout duration decreased with egg-cooling rate (F = 8.69, df = 211, P = 0.004; Fig. 2), increased with clutch size (F = 10.70, P = 0.001), and decreased with embryo age (F = 3.46, P<0.0001).

**Figure 3:**
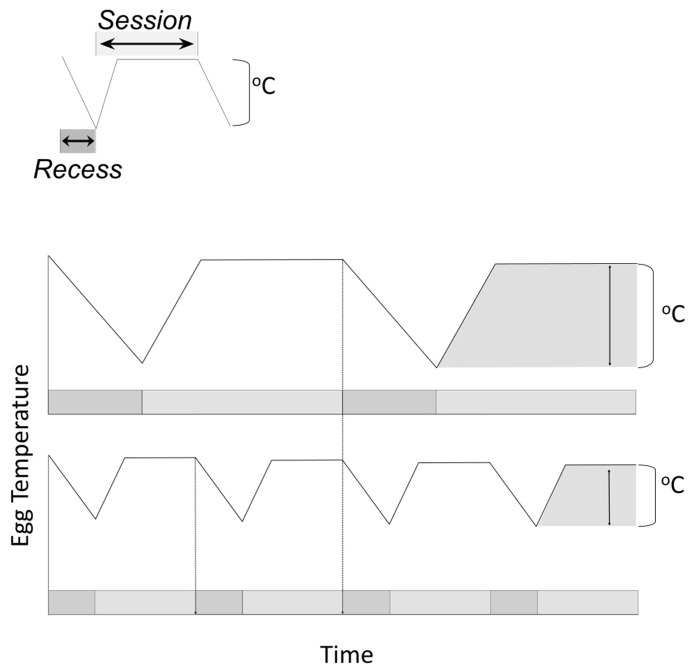
Schematic of egg temperatures resulting from off-bouts/on-bout combinations. Increased frequency of off-bouts from the nest, coupled with a decrease in time on the nest per incubation on-bout, can elevate minimum egg temperature and reduce temperature variations. The key to this counterintuitive result is that incubation on-bouts are not decreased to the same extent as incubation off-bouts. When on-bouts are considered cumulative over the course of a day, nest attentiveness increases in spite of the decreased time on the nest during individual incubation on-bouts. This pattern has the advantage of decreasing temperature variation while simultaneously limiting low egg temperatures.

Females achieved higher nest attendance through a 2.9-fold reduction in the duration of their off-bouts (GLMM: *F* = 3.31, df = 9 and 119, *P* = 0.001), a 1.0-fold increase in the number of off-bouts per day (GLMM: *F* = 3.22, df = 9 and 119, *P*<0.002), and a 1.1-fold decrease in the mean on-bout duration (GLMM: *F* = 2.31, df = 9 and 119, *P* = 0.02). The increased off-bout frequency, accompanied by a reduction in off-bout duration 2.7-times the simultaneous reduction in on-bout duration, resulted in increased daily attentiveness as the incubation period progressed (GLMM: *F* = 1.45, df = 51 and 119, *P* = 0.01; represented in schematic Fig. 3.).

Brood-patch temperatures did not account for changes in egg temperatures; the estimated brood-patch temperature remained constant within the range of 36–42°C (mean 38.5±1.80 SD; *n* = 956) throughout the incubation period, with no significant change with embryo age (GLMM F = 1.98, df = 920, P = 0.16) or clutch size (F = 3.37, df = 920, P = 0.07); this result is consistent with observations for other species [40]. Brood patch temperature did not change as function of mean on-bout duration (F = 0.86, P = 0.3), mean off-bout duration (F = 1.91, P = 0.2), egg-cooling rate (F = 0.12, P = 0.7), or clutch size (F = 0.42, P = 0.5).

### What can we infer about embryo thermal tolerance over the course of incubation?

Mean daily minimum and maximum egg temperatures increased with clutch size (GLMM F = 14.38, df = 234, P<0.0001 and F = 5.24, P = 0.006, respectively), and did not change with embryo age (F = 1.49, P = 0.12 and F = 1.58, P = 0.09, respectively).

**Figure 4:**
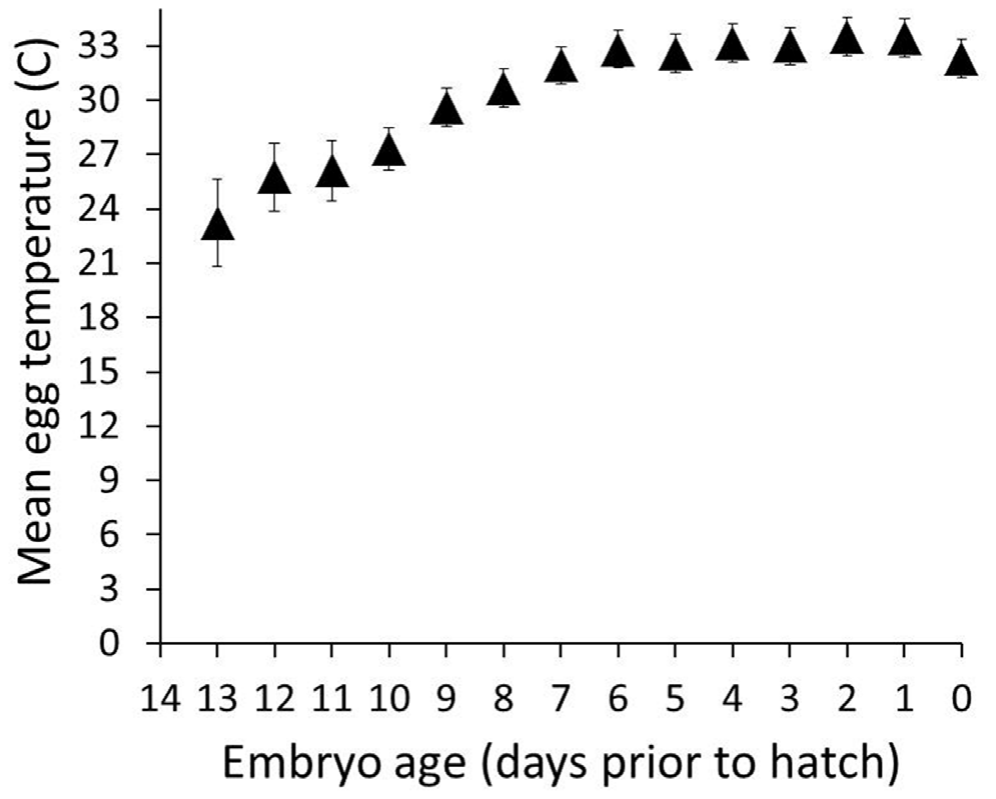
Mean daily egg temperatures. Mean daily egg temperatures (°C) steadily increased over the incubation period until consistently near or above the assumed physiology zero temperature (PZT) of 24–27°C (dashed lines). This pattern is consistent with the hypothesis that embryo thermal tolerances narrow as embryo's age.

Mean daily egg temperature (T_egg_) increased with embryo age (F = 4.86, df = 231, P<0.0001; Fig. 4) and decreased with clutch size (F = 3.21, df = 231, P = 0.04) with no significant interaction (F = 0.85, df = 231, P = 0.68). The coefficient of variation in daily T_egg_ decreased as embryos age (daily T_egg_ coefficient of variation = 8.87±1.35+0.89±0.20×days prior to hatch; df = 36, *F* = 19.63, *R^2^*adj = 0.34, *P*<0.0001). The coefficient of variation in nightly T_egg_ decreased as embryos age (nightly T_egg_ coefficient of variation = 11.7±2.00+1.0±0.29×days prior to hatch; df = 37, *F* = 12.93, *R^2^*adj = 0.24, *P* = 0.001).

## Discussion

Our findings highlight the need to view full incubation as more than a static rhythm; rather, it is a temporally dynamic and finely adjustable parental behavior. Our results are consistent with the idea that physiological changes during embryo development constrain the behavior of intermittent incubators. Egg-cooling rates increased over the course of incubation, consistent with the predictions of the biophysical models [26] [27] and laboratory observations of [28]. Egg-cooling rates had effects at the clutch level, with eggs in smaller clutches cooling faster than eggs in large clutches. Females appeared to adjust incubation rhythms strategically as embryos developed. Despite increasing egg-cooling rates, females maintained eggs at a narrower range of higher temperatures by foraging with short, highly frequent off-bouts. Shorter off-bouts allowed less time for the eggs to cool; this resulted in less temperature variation and eggs spending more time near the maximum egg temperatures. Finally, females took more off-bouts to compensate for the shorter intervals away from the nest.

As embryos approached hatch, the increase in the number of off-bouts for a given day limited incubation on-bout durations; this trade-off resulted in elevated egg temperature through reductions in the durations of their off-bouts and increases in the number of off-bouts per day. In other words, females maintained higher and more constant egg temperatures without having to commit to a *proportional* increase in the overall time on the nest because they adopted a short and frequent foraging strategy (Fig. 3). Although our results clearly show that this species decreased off-bout time *disproportionately* to the decrease in on-bout time, other patterns are possible. A female could, for example, decrease the frequency of incubation off-bouts while simultaneously increasing off-bout duration to allow fewer, longer foraging periods. That pattern would result in extreme decreases in average egg temperature accompanied by greater temperature variation, whereas the observed pattern minimized this.

Our data are consistent with embryo age-related changes influencing female incubation rhythms rather than brood-patch temperature, although other scenarios are possible. The pattern of decreasing incubation on-bout duration as embryos develop is also consistent with the idea that on-bout duration is limited by declining female energy reserves near the end of incubation [22]. Female energy reserves have also been shown to be used more quickly when egg cooling rates are high [41] or when ambient temperatures are low [42]. Many studies of avian incubation operate on the assumption (either explicit or implicit) that females leave the nest to forage when their eggs reach a threshold temperature (e.g., [14]). However, female energetic reserve, not egg temperature, may cue the end of an incubation on-bout [43] [41]. By this scenario, the decrease in the mean duration of incubation on-bouts as incubation progresses and the corresponding increase in cooling rates suggest that females may deplete stored energy levels more quickly late in incubation. Furthermore, the observed patterns of average daily egg temperatures over the course of incubation are consistent with the hypothesis that embryo thermal tolerances narrow as embryo's age.

The decreases in on-bout duration as incubation progressed were also accompanied by increases in mean egg temperature. Although this seems counterintuitive, females limited the time eggs cooled during incubation off-bouts, which had the effect of raising egg temperature even though clutch cooling rates increased, thus maintaining a stable average egg temperature. Our observations are consistent with the assertion [18] that PZT for avian embryos is likely not a set value, but rather shifts over the course of the incubation. Experimental research is needed to confirm whether the changes in female rhythms as embryos develop is likely a response to both increased egg-cooling rate and/or increased embryonic sensitivity to chilling.

The age-specific patterns in egg temperature that we observed in chickadees may be common among intermittently incubating passerines, as they are similar to findings with dusky flycatchers (*Empidonax oberholseri*) and great tits (*Parus major* L.) as summarized by [18]. At the level of ultimate explanations, [18] noted that the patterns were consistent with parental investment increasing as the future reproductive value of the embryos increased with development. At the proximate level, we found patterns that suggest that the narrowing range and higher egg temperatures were shaped by changes in incubation rhythms, rather than by changes in temperature of the brood patch. Thus, we hypothesize that egg-cooling rate is a proximate cue for females to increase investment directly or indirectly. Evidence suggests that brood patches are sensitive to egg temperature [14] and experimental manipulation of egg temperature can alter female incubation behavior [44] [45]. Future experiments manipulating the age of embryos in nests and observing female on-bout and off-bout responses are needed to adequately test this hypothesis under field conditions.

Our findings have implications for studies that examine the conflicting needs of incubating parents and their offspring. The allometric nature of embryonic growth demands that the trade-off between parental self-maintenance and embryo heat requirements be temporally dynamic. Any point in the incubation period at which a parent balances time to both self-maintenance activities and incubation will be brief and transient. Consequently, the portion of the incubation period sampled (e.g., early versus late) will bias any assessment of time allocation between parental energetic requirements and incubation costs.

## References

[1] Clark MM, Galef BGJ (1995) Prenatal influences on reproductive life-history strategies. TREE 10: 151–153. 21236985 10.1016/s0169-5347(00)89025-4

[2] Reid JM, Monaghan P, Nager RG (2002) Incubation and the costs of reproduction. In Deeming DC, editor. Avian Incubation. Oxford: Oxford University Press. pp 314–325.

[3] Larsen VA, Lislevand T, Byrkjedal I (2003) Is clutch size limited by incubation ability in northern lapwings? J Anim Ecol 72: 784–792.

[4] Gorman HE, Nager RG (2004) Prenatal developmental conditions have long-term effects on offspring fecundity. Philos Trans R Soc Lond B Biol Sci 271: 1923–1928. 10.1098/rspb.2004.2799 PMC1691817 15347515

[5] Skutch AE (1957) The incubation patterns of birds. Ibis 99: 69–63.

[6] Deeming DC (2002) Behavior patterns during incubation. In: Deeming DC, editor. Avian Incubation: behaviour, environment, and evolution. Oxford: Oxford University Press. pp 63–87.

[7] Wang JM, Beissinger SR (2009) Variation in the onset of incubation and its influence on avian hatching success and asynchrony. Anim Behav 78: 601–613.

[8] Olson CR, Vleck CM, Vleck D (2006) Periodic cooling of bird eggs reduces embryonic growth efficiency. Physiol Biochem Zool 79: 927–936. 16927239 10.1086/506003

[9] Williams JB (1996) Energetics of avian incubation. In: Carey C, editor. Avian Energetics and Nutritional Ecology. New York: Chapman and Hall. pp 375–416.

[10] Tinbergen JM, Williams JB (2002) Energetics of incubation. In: Deeming DC, editor. Avian Incubation: Behaviour, Environment, and Evolution. Oxford: Oxford University Press. pp 299–313.

[11] Wang JM, Beissinger SR (2011) Partial Incubation in Birds: Its occurrence, function, and quantification. Auk 128 3: 454–466.

[12] Arnold TW (2011) Onset of Incubation and Patterns of Hatching in the American Coot. Condor 113 1: 107–118.

[13] Martin TE, Auer SK, Bassar RD, Niklison AM, Lloyd P (2007) Geographic variation in avian incubation periods and parental influences on embryonic temperature. Evolution 61: 2558–2569. 17714499 10.1111/j.1558-5646.2007.00204.x

[14] White FN, Kinney JL (1974) Interactions among behavior, environment, nest and eggs result in regulation of egg temperature. Science 189: 107–115.

[15] Morton ML, Pereyra ME (1985) The regulation of egg temperatures and attentiveness patterns in the dusky flycatchers (*Empidonax oberholseri*). Auk 102: 25–37.

[16] Hainsworth FR, Moonan T, Voss MA, Sullivan KA, Weathers WW (1998) Time and Heat Allocations to Balance Conflicting Demands during Intermittent Incubation by Yellow-Eyed Juncos. J Avian Biol 29(2): 113–120.

[17] Hainsworth FR, Voss MA (2002) Intermittent incubation: predictions and tests for time and heat allocations. In: Deeming DC, editor. Avian Incubation: Behaviour, Environment, and Evolution. Oxford: Oxford University Press. pp 223–237.

[18] Webb DR (1987) Thermal tolerance of avian embryos: a review. Condor 89: 874–898.

[19] Williams JB (1993) Energetics of Incubation in Free-Living Orange-Breasted Sunbirds in South Africa. Condor 95: 115–126.

[20] Ardia DR, Pérez JH, Chad EK, Voss MA, Clotfelter ED (2009) Temperature and life history: experimental heating leads female tree swallows to modulate egg temperature and incubation behavior. J Anim Ecol 78 1:4–13. 18637971 10.1111/j.1365-2656.2008.01453.x

[21] Haftorn S (1988) Incubating female passerines do not let the egg temperature fall below the “physiological zero temperature” during their absences from the nest. Ornis Scandinavica 19: 97–110.

[22] Wiebe KL, Martin K (2000) The use of incubation behavior to adjust avian reproductive costs after egg laying. Behav Ecol Sociobiol 48: 463–470.

[23] Conway CJ, Martin RE (2000) Effects of ambient temperature on avian incubation behavior. Behav Ecol 11: 178–188.

[24] Ricklefs RE, Starck JM (1998) Embryonic growth and development. In: Starck JM, Ricklefs RE, editors. Avian Growth and Development. Evolution within the Altricial-Precocial Spectrum. Oxford: Oxford University Press. pp 31–58.

[25] Cooper CB, Voss MA, Ardia D, Austin S, Robinson D (2011) Light increases the rate of embryonic development: implications for latitudinal trends in incubation period. Funct Ecol 25: 769–776 doi: 10.1111/j.1365-2435.2011.01847.x

[26] Turner JS (1987) Blood circulation and the flows of heat in an incubated egg. J Exper Zool Supplement 1: 99–104. 3598507

[27] Turner JS (2002) Maintenance of egg temperature. In: Deeming DC, editor. Avian Incubation: behaviour, environment, and evolution. Oxford: Oxford University Press. pp 119–142.

[28] Tazawa H, Okuda A, Nakazawa S, Whittow GC (1989) Metabolic responses of chicken embryos to graded, prolonged alterations in ambient temperature. Comp Biochem Physiol A Physiol 92 4: 613–617. 10.1016/0300-9629(89)90376-9 2566426

[29] Turner JS (1991) The thermal energetics of incubated bird eggs. In: Deeming DC, Ferguson MWJ, editors. Egg Incubation: Its effects on Embryonic Development in Birds and Reptiles. Cambridge: Cambridge University Press. pp 117–147.

[30] Ricklefs RE, Hainsworth FR (1968) Temperature regulation in nestling Cactus Wrens: The development of homeothermy. Condor 70: 121–127.

[31] Mortola JP (2009) Gas exchange in avian embryos and hatchlings. Comp Biochem Physiol A Physiol 153 4: 359–377. 10.1016/j.cbpa.2009.02.041 19285150

[32] Tazawa H, Moriya K, Tamura A, Komoro T, Akiyama R (2001) Ontogenetic study of thermoregulation in birds. J Therm Biol 26 4–5: 281–286.

[33] Smith S (1967) Seasonal Changes in the Survival of the Black-Capped Chickadee. Condor 69: 344–359.

[34] Smith S (1997) *Black-Capped Chickadee*. Stackpole Books. 90 p.

[35] Poussart P, Larochelle J, Gauthier G (2000) The thermal regime of eggs during laying and incubation in greater snow geese. Condor 102 2: 292–300.

[36] Cooper CB, Mills H (2005) Software to quantify incubation behavior from time series recordings. J Field Ornith 76: 352–356.

[37] Voss MA, Hainsworth FR (2001) Relatively simple, precise method to analyze temperature transients in ectotherms. J Therm Biol 26: 121–132. 11163928 10.1016/s0306-4565(00)00032-2

[38] Bakken GS (1976) An improved method for determining thermal conductance and equilibrium body temperature with cooling curve experiments. J Therm Biol 1 3: 169–175.

[39] Littell RC, Milliken GA, Stroup WW, Wolfinger RD (1996) *SAS system for mixed models*. SAS Institute, Cary, North Carolina, USA.

[40] Afton AD (1979) Incubation temperatures of the Northern Shoveler. Can J Zool 57: 1052–1056.

[41] Reid JM, Monaghan P, Ruxton GD (1999) The effect of clutch cooling on starling, *Sturnus vulgaris*, incubation strategy. Anim Behav 58: 1161–1167. 10600136 10.1006/anbe.1999.1241

[42] Bryan SM, Bryant DM (1999) Heating Nest-Boxes Reveals an Energetic Constraint on Incubation Behaviour in Great Tits, *Parus major* . Biological Sciences 266: 157–162.

[43] Chaurand C, Weimerskirch H (1994) Incubation routine, body mass regulation and egg neglect in the blue petrel *Halobaena caerulea* . Ibis 136: 285–290.

[44] Vleck CM (1981) Energetic cost of incubation in the zebra finch. Condor 83: 229–237.

[45] Davis SD, Williams JB, Adams WJ, Brown SL (1984) The effect of egg temperature on attentiveness in the Belding's savannah sparrow. Auk 101: 556–566.

